# Engaging ‘communities’: anthropological insights from the West African Ebola epidemic

**DOI:** 10.1098/rstb.2016.0305

**Published:** 2017-04-10

**Authors:** A. Wilkinson, M. Parker, F. Martineau, M. Leach

**Affiliations:** 1 Institute of Development Studies, University of Sussex, Brighton BN1 9RE, UK; 2 Department of Global Health and Development, London School of Hygiene and Tropical Medicine, 15–17 Tavistock Place, London WC1H 9SH, UK

**Keywords:** community engagement, participation, Ebola, trust, anthropology

## Abstract

The recent Ebola epidemic in West Africa highlights how engaging with the sociocultural dimensions of epidemics is critical to mounting an effective outbreak response. Community engagement was pivotal to ending the epidemic and will be to post-Ebola recovery, health system strengthening and future epidemic preparedness and response. Extensive literatures in the social sciences have emphasized how simple notions of community, which project solidarity onto complex hierarchies and politics, can lead to ineffective policies and unintended consequences at the local level, including doing harm to vulnerable populations. This article reflects on the nature of community engagement during the Ebola epidemic and demonstrates a disjuncture between local realities and what is being imagined in post-Ebola reports about the lessons that need to be learned for the future. We argue that to achieve stated aims of building trust and strengthening outbreak response and health systems, public health institutions need to reorientate their conceptualization of ‘the community’ and develop ways of working which take complex social and political relationships into account.

This article is part of the themed issue ‘The 2013–2016 West African Ebola epidemic: data, decision-making and disease control’.

## 1. Introduction

More than any health emergency in recent times, the West African Ebola outbreak has demonstrated the importance of community engagement and the risks of doing it badly. When the outbreak began in the tri-border region between Guinea, Sierra Leone and Liberia, local ‘communities’ and their ‘traditions’ were frequently portrayed as part of the problem. In the face of a deadly new disease, and an array of suspicious outsiders who were often dressed head-to-toe in protective suits and spraying chemicals, some people chose to cut themselves off from help. They threw stones at ambulances, rioted and, in one episode in Guinea, killed eight members of an Ebola prevention delegation [1]. Reasons for resistance are multiple, ranging from contradictory messaging, unsafe and degrading conditions in hospitals, and histories of violence, extraction and corruption which fed fears that Ebola (or the chlorine disinfectant spray) was a means of ethnic cleansing [2–4]. There has now been damning criticism of the lack of respectful engagement with communities at the outset of the crisis, when the overall approach tended towards shock and blame rather than seeking to understand their misgivings [5,6]. As the epidemic progressed, response tactics changed and the prevailing attitude transformed to one where communities and community-level action was celebrated as central to the response. As Oxfam's ‘Never Again’ report noted, ‘community engagement in the protection and promotion of health has been vital to controlling the outbreak’ [7, p. 10]. The ‘continued mobilization of communities’ is articulated as a principle guiding post-Ebola recovery in Sierra Leone [8, p. 10] and ‘community ownership’ is one of five priority pillars of the Health Sector Recovery Plan [9]. Going a step further, a number of post-Ebola reports suggest community engagement will build trust, a vital ingredient lacking during the outbreak [10–12].

We welcome these more positive reflections on the role of communities and the quality of their engagement during the Ebola epidemic and into the future. Yet, for all the reports extolling the virtue of ‘community engagement’ and ‘community-based’, ‘community-centred’ or ‘community-led’ programmes, there has been less attention paid to what that might entail, and little reflection on the nature of ‘communities’ as locally understood and experienced. This is worrying as there are extensive literatures in anthropology, international development and public health which have emphasized how notions of ‘the community’ can be problematic if used uncritically (e.g. [13–17]). This article is intended to contribute to the reflective process of learning lessons by connecting this literature on communities with ethnographically based insights into how local populations were engaged during the Ebola epidemic. Although ‘community engagement’ improved as time went by, we suggest that it was not as straightforward and complete as it is made to seem in the emerging official histories of the epidemic. We also expose the mismatch between communities as imagined in external interventions and official reflections on them, and the more complex relationships that existed on the ground, and that local populations mobilized in their engagements with outsiders. We argue that lessons about effective ways of engaging with communities in epidemic control will not be learned unless some of the more inconvenient aspects of community-wide approaches are acknowledged and dealt with more clearly.

We start with a vignette that begins to expose these diverse views and experiences of ‘community’. There follows a breakdown of the concept of community and a review of its use in public health, including the challenges involved. We then describe two community-based interventions, one in Guinea and one in Sierra Leone, to illustrate how these issues manifested in interactions between local populations and external agencies during Ebola.

## 2. Community action on burials in practice

In February 2015, one of the authors (A.W.) was invited by a member of the international response to attend a ‘community meeting’ on the outskirts of Freetown. The meeting was called because evidence of a ‘secret burial’ had been uncovered.^1^ Unsafe burials were a source of great tension: medical teams emphasized biosafety to the neglect of families’ spiritual and social obligations [2,18]. ‘Secret burial’ was the term used among response workers when deaths were not reported to the burial teams and when dead bodies were not buried according to the ‘safe and dignified burial’ policy. The term carried with it an aura of mystery and was often accompanied by whispers about ‘secret society’ rituals at Sierra Leone's National Ebola Response Centre (NERC) headquarters. This alludes to the women's and men's initiation societies—in common English parlance, ‘secret societies’—that govern important matters of life and death in the region, and which strictly withhold knowledge of their activities from non-members. The institutions command a mixture of respect, fear and mythologizing by contextual outsiders [19–21]. On this occasion, however, it materialized that a man had put his dead daughter's body down a latrine. Such contextual details were not usually included when the burial data from each district were reported at NERC's punctual 17.00 briefings, perpetuating the gap between response understandings and the experiences of local populations.

At this point in the epidemic a ‘social mobilization pillar’ headed by UNICEF was attempting to coordinate the huge number of national and international partners. There was a focus on making messages consistent, and moving away from mass media and megaphones towards a more engaged approach. Evidence was emerging of local learning and response [22]. A prominent intervention was SMAC (Social Mobilization Action Consortium) who advocated two-way messaging and pursued specific strategies through the medium of radio, and with religious leaders, survivors and ‘communities’. Against this backdrop, some people at NERC were beginning to talk about the possibility of ‘deepened community engagement’ for incidents of ‘non-compliance’ with control measures, as in this particular case of a ‘secret burial’.

So it was that approximately 25 people, a good number of whom were international response personnel, gathered in a small hall on a hillside to the east of Freetown. As it was a peri-urban area, the ‘community’ in question was defined by the administrative unit of the council ward, with ‘key stakeholders’ drawn from there including: the chief, ward counsellor, former ward counsellor, pastor, Imam, original land owner, elders, school headmaster, a junior Community Health Officer, Ebola survivors and the father of an Ebola victim who had been taken to a treatment centre and never returned. This ward had already been engaged through SMAC's flagship Community-Lead Ebola Action (CLEA) campaign. CLEA was rolled out nationally and implemented by two international non-governmental organizations using local staff to facilitate. One of these facilitators was also present. CLEA used participatory methods to ‘trigger’ community responses to Ebola. Similar to many communities who took part in CLEA processes [23], one of the actions this community decided to employ against Ebola were bye-laws, administered by the local chiefdom authorities, forbidding people from burying the dead themselves and fining them if they did so. Most of the meeting consisted of participants lining up to denounce what had happened, to argue for a harsh punishment and insist that the fines were increased to be more of a disincentive. What drove a father—by that point in jail—to put his child's body in a latrine was not discussed and never became clear.

Such meetings were a mainstay of community engagement during the Ebola epidemic. The CLEA programme supported Ebola actions in 60% of communities—villages in rural areas and council wards in urban areas—in each case through public meetings [23]. With its roots in Community-Led Total Sanitation, a highly regarded participatory development approach, CLEA was—on paper—one of the more nuanced mass engagement strategies. The field guide specifies that a cross section of community stakeholders and leaders need to be included. It emphasizes the need to be flexible to ensure that the process is community-led and to look out for quiet and shy people who may be discouraged from participating by those who are more dominant. Yet the CLEA approach hinges on the idea of communities as coherent and cohesive entities. It takes these entities as its ‘unit of analysis’ and promises that Ebola control can be achieved through collective decision-making which ‘builds on social solidarity, cooperation and mutual support’ [24, p. 7]. This picture of communities and community action contrasts starkly with the lack of empathy articulated at the meeting called to discuss the body in the latrine, where the reaction was one of punishment and distance not nurturing dialogue.

While those involved with CLEA, and many others on the frontlines of development and public health, were well aware of the challenges of delivering community-led interventions in practice [23], these challenges do not feature highly in the reports reflecting on the lessons which need to be learned. Instead, there is a tendency to simply state that communities are influential and need to be incorporated in outbreak responses. Given that the supposed cohesive properties of communities are increasingly imagined as a means of building trust, as well as of achieving public health ends, there is a need to critically examine both the concept and the multiple ways in which the term is used.

## 3. What is a community?

There is a well-established literature critiquing the ‘myth’ of community and the problems of applying community concepts [13,15–17,25–27]. Nevertheless, in health and other sectors relevant to development, the term has proved remarkably resilient and it remains widely used by practitioners and social scientists working on public health.

An underlying concern is the lack of clarity about what constitutes a community. Amit & Rapport have suggested that the term is ‘too vague, too variable in its application and definitions to be of much utility as an analytical tool’ [13, p. 13]. Even by 1955, for example, Hillery [28] documented 94 endeavours to define the term, suggesting that the only substantive overlap was that they ‘all dealt with people’ (p. 117). Like other ‘buzzwords’ and ‘fuzzwords’ [29], this vagueness is probably key to the term's pervasiveness. Ambiguity of meaning can allow for disparate actors, with different interests, to seem like they are talking the same language, but it is all the more reason to pay attention to how the concept gets used, and in particular what connotations and intentions are bundled up with it. Critiques have turned in particular on notions of communities as homogeneous, bounded and static, rather than a malleable form subject to changing meanings and representations.

In *Keywords,* Raymond Williams notes the word community has been in the English language since the fourteenth century and has established a range of meanings, from distinguishing between ‘common’ people and those of rank, to people of a certain district or society, to people with common interests or identities [30, p. 75]. Over time, community has come to be ‘warmly persuasive’ [30, p. 76], indicating intimate relationships and politics in contrast to those of the distant formal state. Amit [31] argues that as the world became more globalized and complex, culture and identity have increasingly diverged from place and personal interactions. With this transition, the idea of community as imagined or symbolic has become more prominent (see also [32]).

As Cohen [26] notes, it is thus often helpful to view community as a symbolic construct based on perceived boundaries. In other words, communities and their boundaries are inherently subjective, invisible and exist in the minds of their members or outside observers. While some communities reside in particular geographical localities, this is not necessarily the case and nor is a shared geography automatically indicative of a single community. Furthermore, as the term may refer to a range of social, religious, occupational and other shared characteristics or interests, it is possible for one to belong simultaneously to multiple communities.

## 4. Imagined communities in public health

When it comes to public health, and international development more broadly, a tension arises over these different definitions of community. While health professionals may hold more complex and qualitative senses of community in their minds, institutional constraints and practice often reduce communities to particular geographies, either catchment areas or administrative units, or people living in areas at risk of a particular disease [14]. Such externally generated conceptions may bear little resemblance to local realities, especially in how individuals within those settings regard themselves, and in the multiple identities and relationships that are salient in social and material life. This is problematic because in health interventions the concept of community is not only used descriptively, it is used instrumentally. Interventions to prevent diseases or respond to epidemics require buy-in, support and behaviour change from the people who are at risk. As such, communities are frequently a means to an end, with interventions largely already designed. The addition of positive and inclusive terms such as ‘engagement’, ‘ownership’ and ‘participation’ jar with the realities of programmes which, in effect, roll out preformed plans on populations [17,29]. Simplified notions of community, which gloss over differences, divisions and multiple identities within particular locales, can assist in these objectives or undermine the best of intentions to include local people in the design process [25].

External conceptions of communities and communal life frequently entail projecting uniform patterns of social interaction onto village life, imagining that people live in bounded, predictable units, and even that social relations are harmonious within this space [24]. It is often assumed (or hoped) that community leaders will behave in altruistic ways for the good of ‘their’ community and suggestions of how to engage communities frequently fail to go beyond consulting with ‘community leaders’ [11].

Such imaginaries and logics are problematic for a number of reasons: first, they are premised on the idea that communities are static, unchanging and visible; rather than dynamic, ever-changing and open to context-specific representations [27,33]. Second, they presume social cohesion, taking minimal, if any, account of the heterogeneous array of social divisions and hierarchies and they frequently set aside, or pay minimal attention to, differences (whether by country of origin, occupation, gender, class, caste, age, religion) [17,33]. Power relations are unspecified. This contributes to a third misconception: by overlooking differences and the way in which power relations are embedded in social hierarchies, those scaling up biomedical interventions (such as vaccination campaigns and the mass distribution of drugs for the control of neglected tropical diseases) often mistakenly assume that a ‘one size fits all’ approach will automatically remain effective while benefitting from operational and financial efficiency associated with economies of scale [34,35]. Indeed, it is often suggested that a particular intervention can be rolled out, at speed, without paying attention to the historical, political, economic and social contexts in which it is necessarily embedded.

The literature is replete with examples demonstrating that it is often the most marginal and vulnerable groups who are systematically ignored and not reached by biomedical interventions [35–38]. Not only does this have clear implications for intervention equity, but it also can render transmission control efforts for diseases such as Ebola ineffectual where the continuation of even a single transmission chain has disastrous consequences. Moreover, public health interventions premised on static notions of the community often reinforce or create social hierarchies that are resented, interacting with histories of ill-founded interventions to lead to the rejection of vaccinations, medications and other forms of biomedical care [39]. In some cases, polio vaccination campaigns in northern Nigeria and Pakistan being a stark example, the mistrust generated by an initial failure to take such rejection seriously—resorting instead to naive notions of community engagement and education—can take decades to resolve [39,40]. Additionally, the faith in the desire and capacity of ‘community leaders’ or ‘community health workers’ to work across ethnic, religious and socio-economic boundaries is often ill-founded, especially when their efforts are not remunerated [41]. Meanwhile the involvement of community ‘stakeholders’ and ‘representatives’ in intervention design is frequently regarded as sufficient attention to local sociocultural contexts; yet these people have often been designated by policymakers, or by particular factions among local populations—not an inclusive set of intended beneficiaries. Consequently, the delivery of healthcare to ‘the community’ ends up being very partial indeed.

## 5. The reality of Ebola care and control in communities

As our opening vignette highlighted, trusting relationships and a sense of common interest between people living in geographical areas did not develop uniformly during the Ebola epidemic. There are long-term political, economic and historical reasons why this was difficult [4], but a contributing factor was the way in which policies were rolled out at the community level.

In the Ebola response, the use of the term ‘community’ by public health agencies—and in the representations of some of those who came forward as ‘community leaders’—glossed over a large range of more salient forms of identity, hierarchy and division in social life in the Mano River region. These include gender, in a region where questions of health and sickness are intimately bound up with distinct masculine and feminine realms of knowledge, practice and authority, underpinned by gender-specific initiation societies; lineage, where ‘firstcomer’ and ‘latecomer’ families and individuals hold differentiated authority over land, property and decision-making and positions in patronage systems; age, where elders command the respect and service of youth, linked to control over knowledge and marriage relations; and ethnicity, relating to the historically embedded identities and divisions among people who assemble in any given locality in this highly mobile world. Ethnographic studies in the wider region provide ample detail of such differences, and their multiple significances in everyday life and social and political affairs [21,42–46]; these divisions confounded attempts to intervene in the Ebola epidemic as if communities were unified wholes, echoing the problems identified in wider social science literature.

An illustrative example occurred in Guéckédou, part of Guinea's forest region, in June–July 2014. International response teams had encountered distrust and resistance from local populations, which was contributing to an inability to control the spread of the Ebola virus. As a result, a WHO consultant anthropologist Julienne Anoko was asked to join the response to help understand and allay the tensions [3]. The forest region of Guinea is majority Kissi, an ethnic group with long running experiences of marginalization and abuse at the hands of whites and other ethnicities, first during the French colonial period and then under post-colonial governments. A system of village chiefs and then district officials had been put in place under colonial times, of which people were deeply distrustful. Various response partners trying to contain the epidemic had identified ‘community leaders’ to liaise between them and the ‘community’. Selection had been either through self-identification or assumed from their professional, civic or political associations. Consistent and sometimes violent rejection of outside help, including on one occasion reportedly beating these supposed representatives, was testament to this strategy not working. Trying a different approach Anoko spent three days talking to people and asking them who they would trust and nominate to speak on their behalf. From the long list of names collected Anoko identified those which came up frequently. Comparing this list of names to those the partners had originally been working with, there were none which were on both lists (AW Anoko 2016, personal communication). The second list, spanning 26 villages, contained the names of those deemed to be legitimate representatives and it included: traditional practitioners, heads of the sacred forests, religious leaders (Christians and Muslims), circumcisers, village birth attendants, hunters, youth, returned migrants from the city and elders. A workshop totalling 150 people was organized between people on this second list and response workers allowing each to better understand the other's perspectives and priorities and ultimately lessening the resistance and initiating cooperation [3]. This striking example, where two lists of ‘community leaders’ did not overlap at all, exposes the way externally—or rapidly and naively—generated conceptions of communities, which fail to identify locally recognized sources of legitimacy, authority and influence, can have grave consequences. Indeed, ignoring local politics proved fatal in nearby Womey where eight people partaking in an Ebola sensitization visit were murdered [2].

The implementation of the Ebola Community Care Centres (CCCs) in Sierra Leone reveals further problems with ‘off the peg’ approaches to community, even when they appear to work. The CCCs were conceived at a time of great uncertainty, around September to November 2014, when predictive models were warning of potentially millions of new Ebola cases and there were shortages of beds [47]. In addition, many people were reluctant to be admitted to the few facilities which were available as they were often overcrowded and located far away. CCCs were envisaged as a space closer to, and embedded within, local communities—and thus more locally acceptable—which would enable early isolation of Ebola patients. The UK Department for International Development (DFID) funded at least 54 CCCs in Sierra Leone, with bed capacity ranging from 8 to 25 [48].

There had been fears that residents around the sites where CCCs were to be located would reject them, believing they were bringing Ebola to the area or that they were being used to kill local people. Anticipating this problem, they were designed with a view to being staffed by a mix of trained nurses and local residents, with community meetings to decide on their site. Some CCCs had a community liaison officer whose job was to encourage people in the surrounding area to attend them when they were sick. Thankfully, the statistical models proved unreliable and the worst case scenario did not occur. In fact, the general curve of the epidemic declined before many CCCs opened, meaning that it was rare that they admitted Ebola patients, though they did triage suspect cases and, in some cases, treat other illnesses. This meant one of the biggest tests of community engagement—how people would interpret rising caseloads in their locale—did not materialize.

As documented by Oosterhoff *et al.* [48], the community engagement processes around the CCCs found that people simultaneously appreciated and resented the CCCs. People stated that they liked the service on offer, but they were not satisfied with the way they were set up. In the absence of rising Ebola numbers, and in the face of a normal health system which was not free and, in some places, not functioning due to Ebola, people were pleased to be offered food, medicines and healthcare at no cost. They reported, however, that they had had little say in the planning of the CCCs, with the process being led by response partners, officials and chiefs. The latter were assumed by external agencies to represent their communities—yet villagers often perceived them to have used their influence to ensure that the high salaried jobs went to people under their patronage. The top-down process was felt to be abusive by local people who had often contributed land and labour for free to build the CCCs.

None of this is to say that both the CCCs and CLEA were not formidable achievements given the circumstances. Nevertheless, the extent to which either were modes of engagement based on collective interests needs to be considered. Bye-laws and fines clearly were effective in some areas. Administered through the ‘traditional’ chieftaincy system, they were certainly ‘local’ solutions with historical precedent and legitimacy. This does not mean, however, they were not open to abuse or elite capture. Indeed, there are numerous historical examples of the ambivalent relationship between the chieftaincy system in Sierra Leone and the people they represent. There have been reports of chiefly corruption, for example, in tax collection, and selective use of the law by paramount chiefs and their chiefdom councils [49]. Chiefs have been accused of facilitating large land leases for foreign investors, using threats and violence against labour activists, with promises of local employment opportunities not followed through [50]. The chiefdom system is predominantly gerontocratic, with young people subject to control by elders for whom they perform labour and who control their access to land, citizenship and potential brides [45,51]. It is also predominantly patriarchal, not representing women's interests, with the proviso that certain women of high-status firstcomer families can occasionally reach the rank of paramount chief. Chief-led local courts largely work through the imposition of fines and are often perceived as revenue-generating mechanisms, with justice available to the highest bidder [52]. The implementation of CCCs and bye-laws through chiefs occurred against this background. Although it was efficient in the main, neither came without drawbacks. For example, one Paramount Chief in northern Sierra Leone allegedly earned the nickname ‘PC 500’ for the Ebola fines he was dolling out without obvious disease prevention justification.

In times of emergency, fines, bye-laws and other authoritarian curtailments on freedom, such as quarantines, may be a necessary price to pay for stopping a disease. The point we wish to make here is that trade-offs and difficult decisions will usually be involved and it serves no purpose to romanticize the way community action unfolded. It is more accurate and helpful to say that the CCCs and bye-laws effectively mobilized hierarchical structures of authority, rather than claiming they were ‘community-led’ or ‘owned’. As ever, beneath the apparent consensus of a public meeting there will be unpredictable, secretive and often exclusionary tactics and divisions at play [53]. Unless these realities are noted, lessons will not be learned and trust will certainly not be built.

## 6. Conclusion: learning lessons from Ebola

To a very large degree, the West African Ebola epidemic was brought to an end by large-scale changes in transmission-associated practices and collective action on the part of Mano River populations. Local learning was dramatic, some of it independent and some of it facilitated by externally instigated social mobilization and community engagement efforts [23,54]. The power of people and institutions to learn, adapt and transform in the face of an untreatable disease should be celebrated and a major lesson is the importance of paying attention to the social dynamics and contexts which enable such change.

Using the term ‘communities’ in an uncritical and unreflective way threatens to obscure some of this learning. Many of the most influential post-Ebola reports, which write the history and lessons of the epidemic, praise the role of communities and community engagement. Important action undoubtedly went on at the village, ward and chiefdom level. This paper provides details of the ways in which some key Ebola interventions were not as ‘community’ unifying as these reports make out.

Community is an invitingly non-specific term. It carries a sense of grass-roots collegiality which obscures social complexities and power relations. The labelling of a project as community-based or owned can conceal both the imposition of interventions on people from outside, their implementation through practices which marginalize, or produce resentment and suspicion, and the ability of certain people to use their position amidst social divisions and hierarchies to manipulate and capture interventions to their own ends. Policy options may seem limited in times of emergency. However, rather than relying on externally applied definitions or obscuring uncomfortable realities by continuing to perpetuate imagined qualities of communities, a more constructive response is to find ways to bring sociopolitical orders and relationships more sharply into focus. Efforts should be made to identify the interests of different parties, and to understand the relationships between them and the influences on them, both within the locale and outside of it. This would reveal the structures and strategies which enable change or reinforce existing patterns, and as such provide a better basis for outside interventions.

The lesson from Ebola, then, is not that ‘communities’ can stop epidemics and build trust; it is that understanding social dynamics is essential to designing robust interventions and should be a priority in public health and emergency planning. A critical step is to begin with a more realistic account of local social relationships. Including anthropologists with specialist knowledge of people and contexts in policy formation and implementation can assist this process. A ‘one size fits all’ approach and public meetings with supposed key stakeholders is not enough. To achieve the post-Ebola aims of a trusting public and strong resilient health systems, more nuanced approaches are needed which are sensitive to how social, political and economic interests interact in policy processes and local settings.

## Endnote

1 The meeting was arranged by an employee of the World Health Organization (WHO) who was responsible for social mobilization in Freetown, Sierra Leone. Follow up with this employee in the days after the event and in the process of writing this article, over 18 months later, revealed no further activity or details except that from that point on it was treated as a police matter. A.W. was invited in an informal capacity.
